# Dostarlimab an Inhibitor of PD-1/PD-L1: A New Paradigm for the Treatment of Cancer

**DOI:** 10.3390/medicina58111572

**Published:** 2022-11-01

**Authors:** Faisal K. Alkholifi, Rana M. Alsaffar

**Affiliations:** Department of Pharmacology & Toxicology, College of Pharmacy, Prince Sattam Bin Abdulaziz University, P.O. Box 173, Al-Kharj 11942, Saudi Arabia

**Keywords:** immune checkpoint, PD-1, anti-PD-1 antibody, TSR-042, PD-L1, solid tumors, cancer, dostarlimab

## Abstract

Immunomodulation checkpoints usually adopted by healthy cells by tumors might cause an imbalance between host surveillance and tumor progression. Several tumors are incredibly resistant to standard treatment. The dynamic and long-lasting tumor regressions caused by antibodies targeting the PD-1/PD-L1 checkpoint have suggested a rebalancing of the host–tumor relationship. Checkpoint antibody inhibitors, like anti-PD-1/PD-L1, are unique inhibitors that reduce tumor growth by modulating the interaction between immune cells and tumor cells. These checkpoint inhibitors are swiftly emerging as a highly promising strategy for treating cancer because they produce impressive antitumor responses while having a limited number of adverse effects. Over the past several years, numerous checkpoint antibody inhibitors pointing to PD-1, PDL-1, and CTLA-4 have been available on the market. Despite its enormous success and usefulness, the anti-PD treatment response is restricted to certain kinds of cancer. This restriction can be attributed to the inadequate and diverse PD-1 expression in the tumor (MET) micro-environment. Dostarlimab (TSR-042), a drug that interferes with the PD-1/PD-L1 pathway, eliminates a crucial inhibitory response of an immune system and, as a result, has the potential to cause severe or deadly immune-mediated adverse effects. As cancer immunotherapy, dostarlimab enhances the antitumor immune response of the body.

## 1. Introduction

**Figure 1:**
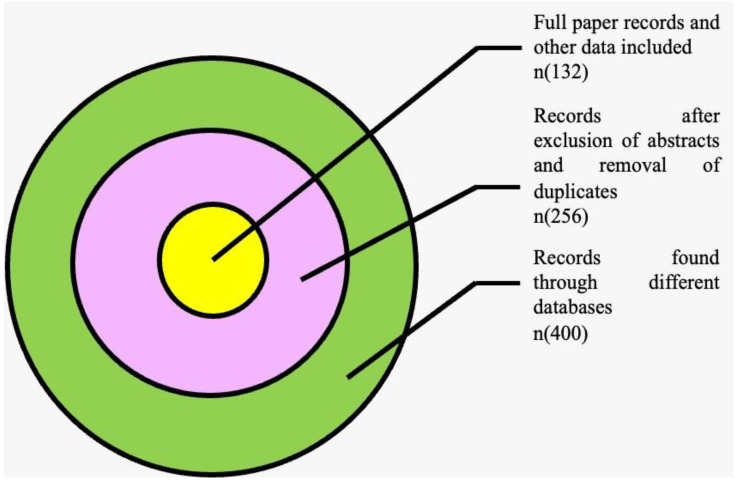
Venn diagram depicting search strategy.

Immune checkpoints have transformed cancer immunotherapy by triggering the immune system. In recent years, new developments have been achieved in the knowledge of the function of the host immune-system tumor development and how it reacts to numerous therapies. One of these exciting new developments in immunotherapy has been a source of great optimism [1]. As a direct result of these developments, novel immunological checkpoint inhibitors have been discovered and approved for clinical use (Figure 1). One of the most successful strategies in cancer-medicine research and development has been the creation of immunological checkpoint inhibitors as prospective anticancer therapeutic alternatives [2]. Inhibitors of immunological checkpoints have become a standard of care for various malignancies, including metastatic melanoma, bladder or urothelial cancer, NSCLC [3], and renal-cell carcinoma. The interaction of (PD-L1)-programmed cell-death ligand 1 and its receptor (PD-1)-programmed cell death 1 suppresses the immune response and is necessary for self-tolerance, avoiding autoimmunity, and immunological dodging [4,5,6,7,8]. They are now being tested for various cancers, like advanced solid cancer, breast cancer, neck and head cancer, and hematological cancers. The hammering of immune regulation has been highlighted as one of the mounting characteristics of cancer [9]. In 1996, Leach et al. anticipated immunological checkpoint inhibition as a novel cancer therapy [10]. Ipilimumab, also known as an anti-CTLA-4 (cytotoxic T-cell lymphocyte-liked protein 4) antibody, was the first immune checkpoint inhibitor authorized by the FDA to treat melanoma in 2011 [11]. This development laid the groundwork for immunotherapy in cancer treatment [5,12,13]. At this time, the FDA has given approval for clinical use to two different types of immunotherapy: (1) inhibitors of either the PD-1 or its PD-L1, and (2) CTLA-4 [14].

Cancers with dMMR mutations have an increased expression of the T-cell proteins PD-1, PD-L1, and PD-L2, which, when activated, limit T-cell proliferation and cytokine production. They interact with PD-1, an immunological checkpoint that dampens the antitumor response of the immune system [15]. The cellular immunity mediated by T cells is tightly maintained and regulated by a check/balance mechanism that relies on various inhibitory and stimulatory proteins. Inhibitory receptors, also named immunological checkpoints, modulate the activation and effector activity of CTL cells to maintain self-tolerance and reduce the harm done to bystander tissues as a consequence of an immune response to a pathogen [1]. Immune-checkpoint medicines that target PD-1 and PD-L1 may reactivate cytotoxic T cells to combat cancer cells. These ligands can bind to PD-1, an immunologic checkpoint inhibiting the immune response against tumors. When a T cell’s TCR recognizes antigens in MHC, checkpoint molecules control the signaling of costimulatory factors like CD-28 to magnify the signal, whereas co-inhibitory molecules reduce it. The expression of immune-inhibitory checkpoints like CTLA-4 and PD-1/PD-L1 has recently been proven to be an effective mediator for controlling and evading phases of cancer-immune editing [16]. When these molecular components bind to ligands on APCs, they dampen the anticancer response. Recent attempts to employ mAbs to target and disrupt immune-inhibitory interactions specifically have ushered in a new era of cancer-immunotherapy medications [17,18,19]. In recognition of their work in developing an anti-immunomodulatory treatment for cancer, James P. Allison and Tasuku Honjo were conferred the Nobel Prize in Physiology or Medicine on 3 October 2018. Their study on the immunological checkpoints PD1 and CTLA-4 indicated that they played a “brake” role in immune activity and recommended that hindering immune checkpoints might reawaken T cells and more efficiently eradicate cancer cells [20]. According to many studies, immune-checkpoint inhibitors may have considerable therapeutic potential.

The primary objective of contemporary immunotherapies for cancer treatment is to shift the equilibrium away from a microenvironment that is favorable to tumor growth and toward one that is hostile to tumor growth. Consequently, this enables the immune system to mount the most effective possible response to tumors. In addition, a significant emphasis is being placed on negative regulatory mechanisms. Ipilimumab for advanced melanoma, and patients with castrate-resistant prostate cancer [21] and Nivolumab evaluated in renal-cell cancer, NSCLC, colorectal cancer, and melanoma, all evidenced anticancer activity in phase I/Ib studies prior to receiving FDA consent for different cancers [22,23]. MK-3945 (pembrolizumab) is a humanized antibody [24,25,26]. Before their most recent FDA clearance for many malignancies, the anti-PD-L1 mAbs atezolizumab and durvalumab demonstrated preclinical anticancer efficiency in various solid tumors [27,28,29,30,31,32]. Finally, dostarlimab is a mAb that hooks to the PD-1 receptor and snags interactions with PD-L1 and PD-L2, enabling the antitumor immune response to continue unchecked [33]. Dostarlimab has been shown to be operative in numerous cancers, including mismatch repair-deficient pan malignancies, second-line mismatch repair-deficient endometrial cancer, and NSCLC [34,35,36]. The SHM-XEL system from AnaptysBio was employed to design dostarlimab. This approach consisted of gluing the heavy- and light-chain complementarity-determining sections onto the germline-variable region scaffold of their most immediate orthologs from the human species, tailed via affinity maturation via somatic hypermutation and mammalian-cell display [37,38]. Dostarlimab single-agent anticancer efficacy was assessed using humanized mice models because of the dearth of cross-reactivity with mouse PD-1. In this model system, the anticancer activity of dostarlimab was demonstrated by the reduction of tumor development, which was associated with augmented inflammatory cell infiltration. These results demonstrate the potency of dostarlimab as an anti-PD-1 receptor opponent with properties that call for additional clinical trials in cancer patients. Dostarlimab, marketed under the trade name Jemperli, is presently being investigated in several different solid tumor types as part of the phase I GARNET trial (NCT02715284), which is both a dosage acceleration and a safety/adequacy cohort expansion research [39,40]. Dostarlimab is certified as monotherapy for diagnosing adult patients with reoccurring or advanced mismatch repair deficiency/microsatellite instability–high endometrial cancer that has advanced while receiving a platinum-containing regimen or following treatment that consisted of platinum in the past. Dostarlimab has been showing antitumor activity that is clinically relevant, with disease-control rates of 57.7%, objective response rates of 42.3%, and safety profiles that are commensurate with those of licensed anti-PD-1 medicines [38,41]. The FDA has given its approval to a number of medications that target PD-1/PD-L1, and more investigation is still being conducted (Table 1).

## 2. Methodology

A search of the PubMed, EMBASE, and Cochrane databases was performed separately and individually using keywords such as immune checkpoint, PD-1, TSR-042, PD-L1, anti-PD-1 antibody, solid tumors, cancer, and dostarlimab until August 2022. Clinical-trial registries/databases and websites were checked for relevant information. Any disputes amongst the authors were resolved after a conversation. We also looked at the studies’ citations and previous meta-analyses to see whether they qualified. Research eligibility was determined based on the study’s title and abstract. In the second step, the whole texts of qualifying papers were scrutinized before they were excluded from consideration. According to the specified keywords, there were 400 results. Two hundred fifty-six publications were chosen after screening and used in the subsequent investigation (Figure 1). One hundred thirty-two articles, including clinical-trial data, were selected for qualitative analysis after removing duplications and some abstracts.

## 3. K3. Overview of PD-1/PD-L1 Signaling Pathways in Cancer

**Figure 2:**
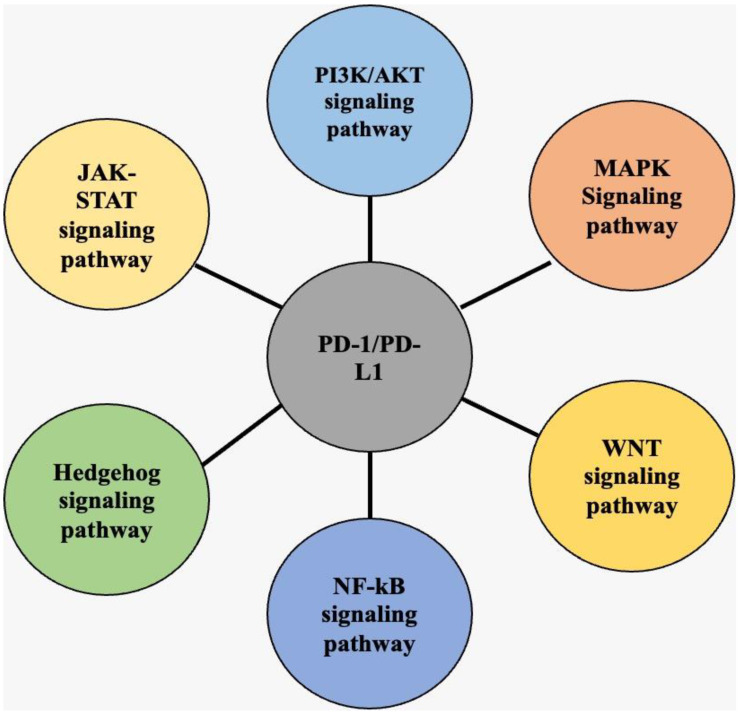
Depiction of different pathways that regulate expression of PD-1/PD-L1. The PI3K/AKT pathway, WNT pathway, MAPK pathway, NF-κB pathway, JAK/STAT pathway, and Hedgehog (Hh) pathway stimulate the expression of PD-1/PD-L1 axis.

The latest research has given researchers improved knowledge of the components that suppress the anticancer immune response, ensuing in the development of various medicines that target immunological costimulatory and inhibitory checkpoint pathways. The PD-1/PD-L1 axis, which plays a crucial role in carcinogenesis, may be regulated by various signals in cancerous cells (Figure 2). Therefore, it is crucial to study this signaling network to advance the current understanding. Tumor-inducing advanced checkpoint molecules mediate antitumor immune suppression like PD-1. The PD-1/PD-L1 pathway adversely controls the immune system in standard conditions [5]. When PD-1 adheres to PD-L1, it is phosphorylated and subsequently increases immunological suppression by activating some intracellular pathways, which is an important location for PD-1’s biological actions [50]. It is noteworthy that PD-1 affects T and B cells differently via different specific pathways [51]. The PD-1/PD-L1 pathway arose physiologically due to the requirement to limit the level of inflammation at antigen-expressing sites to protect healthy tissue from harm. There is a high expression level of PD-1 on the exterior of all activated T cells [52]. An inflammatory recognizes that it is triggered when a T cell identifies an antigen presented by the MHC complex on a target cell. This results in the production of pro-inflammatory cytokines. The PD-1/PD-L1 axis, which is very important in the process of carcinogenesis, may be modified in cancer cells by various signals. Consequently, it is essential to maintain vigilance over this signaling network to advance our understanding (Figure 2). On the cell membrane, PD-L1 is strongly expressed by cancer cells [53]. Cancer cells can dodge immune surveillance and killing because of the negative signals produced when PD-L1 binds to PD-1 in T cells. These signals cause T cells to die and decrease immunocompetence. Furthermore, the differentiation of memory T cells (Tm) and effector T cells (Tef), as well as the differentiation of exhausted T cells (Tex) and regulatory T cells (Treg), is negatively impacted by the activation of the PD-1/PD-L1 pathway, both of which significantly inhibit the immune effect of T cells [54]. By causing the release of cytokines and cytotoxins, the interaction of PD-L1 to PD-1 also prevents the growth of tumor-specific T lymphocytes and triggers death [55]. Cancer cells can also use the circulatory system to carry PD-L1 (carried in exosomes) to distant areas. Therefore, before they reach metastatic tumors, they can remotely suppress T-cell activation [56,57,58]. By stimulating PTEN, PD-1 prevents TCR-induced activation of the PI3K/AKT pathway [59]. In addition, PD-1 reduces T-cell proliferation by preventing the RAS-MEK-ERK pathway from being activated [60]. IFN-γ and IL-2 production by T cells is reportedly decreased by PD-1 because it prevents PKC from becoming activated [61]. Moreover, by inhibiting glycolysis and encouraging fatty-acid and oxidation lipolysis, PD-1 signaling controls T-cell metabolism [62]. 

### 3.1. Programmed Death 1 (PD-1)

PD-1 belongs to the immunoglobulin superfamily PDCD1 gene. Ishida et al. found and published it for the first time in 1992 [55,56,57,63,64,65]. PD-1 is a cell-surface receptor and is expressed selectively on cells that are undergoing apoptosis [66,67]. Eventually, PD-1 was recognized as the primary immunological checkpoint for controlling the thresholds of T-cell and B-cell responses to antigens. As a crucial gatekeeper for T lymphocytes, PD-1 regulates their biological activity extensively [67]. The connection between PD-L1 and PD-1 suppresses T-cell activity by generating T-cell fatigue, promoting immune evasion. Consequently, immune dodging is caused by excessively elevated PD-L1 levels in some immune cells and cancer cells. Antibodies against PD-1/PD-L1 have been a trending issue in cancer immunotherapy. PD-1 shares a similarity of 15% with an amino-acid sequence of CD-28, 20% with CTLA4, and 13% with triggered T-cell co-stimulator [68]. PD-1 is a 288-amino-acid transmembrane protein that is 55 kDa in size and has an extracellular N-terminal domain (IgV-Like), a membrane-permeable domain, and a cytoplasmic tail at its N and C termini with two tyrosine bases each [69]. When PD-1, a blocker of both adaptive and innate immune responses, is expressed by activated natural-killer T cells, macrophages, CD4+ T cells, CD8+ T cells, B cells, monocytes, and DCs, its production is stimulated by the T-cell or B-cell receptor pathway and increased by the stimulation of TNF [68,69,70]. Nevertheless, PD-1 is only weakly expressed by naïve T and B cells [71,72]. Transcription factors like NOTCH, NFAT, IRF9, and FOX O1 may elicit the transcription of PD-1 [73]. The PD-1 gene’s expression is regulated by the conserved upstream regulatory regions B and C. Due to its demethylated promoter, PD-1 is produced in fatigued TCD8 cells during persistent infections, and the FOXO1 transcription factor attaches to the PD-1 promoter to boost its production [74]. Leakage from cancer cells raises the expression of the AP1 component c-FOS, which raises the expression of PD-1 [75]. PD-1 has two roles since it may serve both positive and harmful purposes. Regarding its advantages, it helps to keep immunological tolerance and reduce ineffective or harmful immune responses. However, PD-1 encourages the expansion of cancerous cells by impeding the defensive response of the immune system [76].

### 3.2. PD-L1

The PD-1 ligand, also named CD279 and B7-H1, is a type 1 transmembrane glycoprotein with a 33-kilodalton size, 290 amino acids, and IgC and Ig domains in its extracellular region [77,78]. It is common for PD-L1 to be expressed by DCs, macrophages, activated B and T cells, and specific epithelial cells, predominantly under inflammatory situations [79,80]. Furthermore, tumor cells express PD-L1 as an “adaptive immune strategy” to evade anti-cancer responses [70]. According to observations, tumor-infiltrating DCs show a high amount of PD-L1, essential for inhibiting antitumor immune responses. In a mouse model devoid of PD-L1 on DCs, the therapeutic impacts of PD-L1 blockage vanish entirely. Following antigen presentation, PD-L1 is increased on DCs to shield them from the cytotoxicity of activated T lymphocytes. However, this action also inhibits the immune system’s anticancer responses. These findings also expand our understanding of the processes behind immune-checkpoint blockade treatment [81]. Studies have successfully shown that DC 32-induced intrinsic T-cell tolerance (deletion and/or anergy) depends on the PD-1 pathway. However, constitutive overexpression of these inhibitory molecules led to lower IL-2 generation by T cells amid the high expression of PD-L1 and PD-L2 by mature bone marrow-derived DC, showing that altering the ratio of stimulatory and inhibitory signals affects T-cell activation. PD-L1 has been linked to a high concentration of CD8+ T cells, the production of Th1 cytokines and interferons, chemical factors, and specific patterns of gene expression [71]. The IFN-γ secreted by NK cells activates the JAK1, JAK2, and STAT-1 pathways, which causes tumor cells to produce the PD-L1 on their surfaces [82]. Furthermore, IFN-γ produced by T cells via the JAK1/JAK2-STAT1/STAT2/STAT3/IRF1 pathway has been demonstrated to control the expression of PD-L1 in melanoma cells. IFN-γ seems to be secreted by T and NK cells, which causes PD-L1 expression on the target cell’s surface, which includes cancer cells [83]. PD-L1 acts as an element that promotes the development of tumors because it establishes connections with its receptors and activates the signaling pathways for proliferation and survival. These findings also showed that PD-L1 could play a part in tumor development [84]. It has been discovered that PD-L1 has non-immune proliferative effects on several different kinds of tumor cells. For instance, in renal cancer cells, PD-L1 activates epithelial-to-mesenchymal transition (EMT), which indicates the activation of the PD-intrinsic L1 pathway [85].

### 3.3. PI3K/AKT Signaling Pathway

It has been demonstrated that stimulation of the PI3K/AKT pathway increases the consumption of nutrients and energy generation of CD8+ T cells, and mTOR is an essential factor in regulating the physiological repercussions of immune-cell stimulation [86]. When PI3K/AKT is active, an increase in extrinsic signaling or a reduction in the expression of negative regulators like phosphatase and tensin homolog (PTEN) can stimulate an escalation in PD-L1 synthesis. Downregulation of PTEN might activate PI3K/AKT, making it possible to promote PD-L1 expression [87]. In addition, Zhao et al. discovered that inhibiting PD-1/PD-L1 prevents the death of CD8+ T cells in gastrointestinal stromal tumors (GIST) by modulating the AKT/PI3K/mTOR pathway. PD-1/PD-L1 serves a significant function in the immune system in connection with the PI3K/AKT/mTOR network. Additionally, it has been demonstrated that PD-L1 knockdown in GIST cells mitigates the expression of p-AKT and p-PI3K [88]. Furthermore, Wei et al. found that overexpression of PD-L1 in colorectal cancer cells activated PI3K/AKT in the nucleus [89].

### 3.4. MAPK Signaling Pathway

The MAPK signaling pathway is an essential component of the signal-transduction system that converts extracellular signals into responses within the cell. Phosphorylation activation may also be a mechanism via which it controls cell proliferation, metastasis, differentiation, invasion, and death [90]. Three parallel routes make up the MAPK pathway [91]. These pathways are called c-Jun amino-terminal kinase, ERK, and p38 MAPK. Recent studies have steadily concentrated their attention on the connection between the MAPK pathway and the PD-1/PD-L1 axis. For instance, Stutvoet et al. illustrated that suppression of the MAPK pathway precluded epidermal growth factor and IFN-γ induced CD-274 mRNA and PD-L1 protein and membrane up-regulation in lung carcinoma cells [92]. Furthermore, Jalali et al. observed that the PD-L1 antibody is related to the MAPK signaling molecules in Hodgkin’s-lymphoma cells (HL). It was also discovered that p38 and p-ERK were lowered in all HL lines after employing an anti-PD-L1 antibody. These findings were based on the fact that the PD-L1 antibody is associated with MAPK signaling molecules in HL cells. Likewise, suppression of MEK1/2, a key component of the MAPK pathway in renal-cell carcinoma, can significantly reduce PD-L1 expression [93].

### 3.5. JAK-STAT Signaling Pathway

Activating STAT pathway by JAK signaling is an evolutionarily conserved signaling mechanism exploited by several IFNs, cytokines, growth factors, and other substances [94]. This method lays the groundwork for a crucial regulatory mechanism for gene expression influenced by environmental variables. Consequently, it might be utilized as a fundamental notion for how cells react to environmental factors and elucidate signals to govern the growth and differentiation of cells [95]. The JAK/STAT pathway has recently been found to promote PD-L1 expression in cancer cells that might be helpful in cancer treatment. AG490, an inhibitor of JAK2, inhibited PD-L1 increase at both the protein levels and mRNA, according to Toshifumi et al. [96]. These studies indicated that the JAK/STAT pathway controls PD-L1 expression. In addition, fibroblast growth-factor receptor signaling effectively stimulated the JAK/STAT3 signaling pathway in vitro, leading to elevated PD-L1 expression. Tumor development was accelerated in CRC xenograft models when FGFR2 was overexpressed, and PD-L1 expression was also increased. Inhibitors of the JAK/STAT3 pathway can alleviate this effect [97].

### 3.6. WNT Signaling Pathway

Malignant transformation, tumor development, and resistance to traditional cancer treatment have all been linked to deregulating WNT signaling [98]. Abnormal Wnt signals have also been shown to affect cancer immunomonitoring, encouraging immune escape and resistance to several immunotherapies, such as immune-checkpoint blockers [99]. Active signaling from the GSK3B–TrCP axis, for example, aided the proteasomal deprivation of non-glycosylated PD-L1 in mice models of breast cancer (e.g., without WNT ligands). GSK3 blockers, on the other hand, promote tumor elimination in animal melanoma models by suppressing the PDCD1 gene [100]. Furthermore, functional interaction between WNT activity and PD-L1 expression underpins specific WNT activators or inhibitors to lower or enhance PD-L1 expression for the cure of triple-negative breast cancer (TNBC) [101].

### 3.7. NF-κB Signaling Pathway

Recent research has revealed that the expression of the PD-L1 gene may be influenced by either the IFN-driven nuclear factor (NF)-κB or the Toll-like receptor (TLR). Breast cancer, colon cancer, and melanoma cell lines were observed to grow less rapidly when curcumin, an NF-κB inhibitor, was combined with anti-CTLA-4 checkpoint suppression therapy [102]. Based on this discovery, it is likely that the inhibition of NF-B may have a dual function in the multiplication and durability of tumor cells and tumor immune checkpoints. Caffeic acid phenethyl ester, an NF-κB inhibitor, inhibited PD-L1 induction, indicating that NF-κB is tangled in LMP1-induced PD-L1 expression. NF-κB also mediates INF-B-induced PD-L1 expression. IFN-induced PD-L1 expression was reduced by NF-κB inhibitors but not by PI3K, MAPK, or STAT3 inhibitors [103]. Furthermore, Peng et al. showed that chemotherapy causes local immune inhibition in ovarian cancer through NF-κB-mediated PD-L1 overexpression [104].

### 3.8. Hedgehog Signaling Pathway

Recent advances in therapeutics have mostly concentrated on the research and production of small-molecule inhibitors like SMO and GLI that modify the composition of the Hedgehog (Hh) signaling pathway [105]. It is now understood that the Hh signaling route is necessary for proliferation of substrate cells; furthermore, abnormalities in this system might result in the improvement of tumors. It has been determined that Hh signaling may affect PD-L1 expression, and it has been revealed that blocking Hh signaling can result in lymphocytes exhibiting anticancer activity [106]. According to Jayati et al., Hh signals have a role in developing PD-L1 expression in gastric cancer. The inhibition of PD-1/PD-L restores Gli2-induced tolerance, combined pharmaceutical therapy suppresses Hh signaling, and immunological checkpoints may be beneficial for certain patients [107].

## 4. Overview of the Role of PD-1 and PD-L1 in Cancer

A recent study has shown that EMT is related to the expression of PD-L1 in human breast-cancer stem cells. Likened to the cell line, PD-L1 was shown to be increased in BT-549 and MCF-7 (cell lines) tumorspheres, which is partially dependent on the demethylation of the PD-L1 promoter. Pathological investigations have revealed that PD-L1 levels are more significant in numerous estrogen receptor-negative breast-cancer subtypes. Liu et al. observed that the average level of PD-L1 mRNA was significantly lower in estrogen receptor-positive breast-cancer cell lines than in ER-negative breast-cancer cell lines. Recent studies have revealed that PD-L1 slows the development of cancer cells and that silencing PD-L1 results in an upsurge in spontaneous and doxorubicin-induced apoptosis in breast-cancer cells [108]. Daniel et al. anticipated that anti-C5a medication might improve the capacity of PD-1 to inhibit the development of lung cancer. In the subcutaneous 393P model, the anti-C5a l-aptamer AON-D21 and anti-PD-1 monoclonal antibody RMP1-14 suppressed tumor development more effectively than either therapy alone [109]. In lung cancer, Kazuki et al. revealed that PD-L1 was positively linked with male gender, advanced stage of cancer, smoking, vascular invasion, EGFR gene alterations, and squamous-cell carcinoma histology. According to multivariate and univariate survival assessments, PD-L1-positive individuals exhibited a more unsatisfactory prognosis than PD-L1-negative patients [110].

Furthermore, new studies indicate that PD-1 molecules are favorably expressed on the membrane of Lewis lung-cancer cells, T lymphocytes of C57BL/6 mice spleen, and peripheral blood T lymphocytes. PD-L1 expression can aid tumor cells in evading immune surveillance and boost Treg activity in CRC. Expression of PD-L1 is more rampant in metastatic CRC than in main CRC, and PD-L1 expression in primary CRC may not indicate cancers that have migrated to distant organs. The expression of PD-L1 in metastatic CRC should be taken into account separately for determining patient eligibility for immunotherapy [111]. Patients with gastric cancer who had EBV infection are more likely to have PD-L1 (*p* = 0.009) and TIIC. PD-1 and PD-L1 expression is a favorable prognostic marker that indicates the dosage effect on the mortality of gastric cells. According to the findings of Wang et al., pharmacological inhibitors and small interfering RNA may both inhibit edphatoe, which in turn can raise the levels of PD-L1 in cultivated gastric-cancer cells and xenografts. The degree of PD-L1 expression has been demonstrated to be associated with the intensity of bladder cancer. Zhu et al. revealed that PD-L1 is controlled by the ATG7 autophagy protein in bladder cancer and that overexpression of ATG7 elevated PD-L1 protein levels primarily through boosting autophagy-mediated deprivation of FOXO3a [112]. These results demonstrated that a combination of autophagy inhibitors and PD-1/PD-L1 could increase the effectiveness of BCG in people [97]. D-L1 has been deemed a novel prognostic indicator for pancreatic-cancer patients. It has been hypothesized that inhibiting PD-L1 can effectively decrease pancreatic cancer in mice models by boosting IFN-r and decreasing IL-10 [113]. An additional investigation revealed that the PD-1 expression on peripheral CD8+ T lymphocytes was significantly greater in pancreatic ductal adenocarcinoma patients than in intraductal papillary mucinous neoplasm patients or healthy donors. An ROC investigation additionally validated the diagnostic value of PD-1 expression in PDAC. The expression of PD-1 on a peripheral CD8+− T lymphocyte correlates considerably with the clinical stage, N, and M classification of PDAC [114]. Current research indicates that pD-1, PD-L1, and PD-L2 are expressed at greater levels than Il-17rc in the prostates of Il-17rc wild-type mice. This study suggests that an increase in PD-1/PD-L1/2 expression might boost immune suppression within the tumor microenvironment, favoring prostate-cancer development. PD-L1 is expressed differently in patients with primary prostate cancer [115]. 

Moreover, elevated expression of PD-L1 has been associated with an unsatisfactory prognosis [116]. Therefore, PD-1/PD-L1-targeted diagnosis may be a viable treatment preference for patients with hormone-naive prostate cancer. In addition, PD-L1 is expressed in the cancer cells of DLBCL and the non-cancerous cells that invade the tumor. On the other side, the expression of PD-1 on tumor-infiltrating lymphocytes (TILs) in DLBCL patients has been related to improving overall survival (OS) [117]. It has been proposed that the PD-L1 protein plays a critical role in the tumor microenvironment of DLBCL patients, leading to a more destructive clinical phenotype and a worse prognosis [118] that is not typical of GCB patients (e.g., suppression of T-cell proliferation and IFN-*γ* production by tumor-associated T cells). Patients with EBV-related DLBCL may be prime candidates for anti-PD-1/PD-L1 immunotherapy, as PD-L1 and DLBCL are also associated with EBV infection [119].

### 4.1. PD-1/PD-L1 Inhibition and Their Inhibitors

PD-1 and PD-L1 play a significant role in inhibiting the effector activity of T-cells [1]. Targeting PD-1 and PD-L1 is a potent method for maintaining the function of effector T-cells. Checkpoint inhibitors are mAbs that interrupt the interaction between PD-1 and PD-L1 and eradicate the drawbacks of standard anti-cancer treatment. Lussier et al. conducted research in both in vitro and in vivo settings, and their results demonstrated that inhibiting PD-1 using an antibody can substantially improve the activity of T cells [120]. In addition to their ability to decrease solid tumors inhibiting advanced malignancies and metastasis and usually lengthening patients’ lives, mAbs can considerably reduce toxicity while remaining well below acceptable limits [121,122]. Several anti-PD-1 and PD-L1 mAb clinical studies are currently developing. Some of these have progressed to phase III clinical studies, which are beneficial to many patients. The FDA has recently approved anti-PD-1 and PD-L1 mAbs targeting a number of human malignancies. The clinical efficacy of anti-PD-1 and PD-L1 mAbs offers a promise to focus on PD-1 and PD-L1 immune checkpoints, considerably improving the conditions of patients [123]. The FDA-approved anti-PD-1 treatments and ongoing clinical studies for renal-cell carcinoma, HNSCC, NSCLC, and bladder cancer are outlined in Table 1. PD-1 and PD-L1 are receptor-ligand systems, and in the tumor microenvironment they bind to one another, inhibiting anti-tumor immune responses. The T cells of the immune system are where PD-1 is most abundantly produced; however, PD-L1 is found on cancer cells and APCs. Hence, inhibitors that prevent the contact between PD-1 and PD-L1 will restore T-cell-mediated antitumor immunity [50,104]. PD-1 and PD-L1 antibody inhibitors suppress either PD-1 or PD-L1 and activate T-cell-mediated immunity. It is unclear whether PD-1 or PD-L1 blockers are more efficacious now. The efficacy of PD-1 and PD-L1 inhibitors is dependent on patient factors, like (i) gender, (ii) kinds of cancers, (iii) translocation and gene mutation (Kras, EGFR, ALK), and (iv) tumor metastases [124]. As tumors are diverse, the expression of PD-L1 is not consistent; hence, PD-L1 immunohistochemical labeling differs between tumor regions. As a result, the role of PDL1 expression and PD-L1 inhibitors in determining whether a patient responds to treatment remains a hotly debated topic [125]. There have been promising results in phase I studies using anti-PD-1 medications like nivolumab and pembrolizumab in patients with NSCLC, metastatic melanoma, or renal-cell carcinoma, as well as other solid tumors. Patients with advanced melanoma responded better to PD1 blockers in phase III studies than patients with NSCLC and RCC, which were both evaluated in phase I. As a result, nivolumab has been authorized by the FDA as a first- and second-line therapy for metastatic melanoma, RCC, and squamous NSCLC. Pembrolizumab is also the initial therapy choice for patients with metastatic NSCLC and metastatic melanoma [126]. Atezolizumab, the first FDA-approved PD-L1 inhibitor, has been utilized as first-line therapy for cisplatin-resistant metastatic NSCLC and metastatic urothelial carcinoma. Several clinical studies are underway for gastrointestinal malignancies like renal-cell, colon, urethral, and head and neck cancer [31,127,128].

### 4.2. Resistance Mechanisms

Anti–PD-1/PD-L1 antibodies have demonstrated significant efficacy; however, their advantages are confined to a subset of patients. In other words, most patients demonstrate initial resistance to immune-checkpoint blockers [129,130]. Even once responses are established, a patient’s tumors will ultimately develop a resistance mechanism to evade the immune system. According to results from a biomarker biopsy study conducted as part of the ongoing phase I investigation of MPDL3280A, patients who reacted to therapy were more likely to have tumor-infiltrating cells and tumor PD-L1 expression to begin with [131]. On the contrary, relatively few CD8+ T cells were detected in the tumor’s periphery in a PD-L1-negative patient who did not react to treatment. In addition, a gene-expression investigation indicated no expression of cytotoxic T-cell markers after treatment, suggesting that there was little infiltration of T cells [132,133].

## 5. Jemperli™, Dostarlimab-Gxly (Dostarlimab)

A monoclonal anti-programmed death-1 antibody called JemperliTM, dostarlimab-gxly (Dostarlimab) is produced by GlaxoSmithKline (GSK) with permission from AnaptysBio Inc., San Diego, CA, USA, for the diagnosis of multiple cancers, which include ovarian cancer, endometrial cancer, head and neck cancer, peritoneal cancer, pancreatic cancer, and malignant melanoma. It has been demonstrated that the immune-checkpoint receptor known as PD-1 can inhibit immune responses directed specifically towards cancer [134]. Dostarlimab is a mouse hybridoma-derived humanized IgG4 monoclonal antibody. In order to function, it binds to a PD-1 receptor and inhibits its function. Additionally, it blocks the ligands (PD-L1 and PD-L2) from interacting with the receptor, which activates T cells and restores immune function. Dostarlimab is approved for use in the treatment of adult patients with mismatch repair deficiency (dMMR) and recurrent or advanced endometrial cancer in the United States of America [135,136] and the European Union [137,138]. Dostarlimab, marketed by GlaxoSmithKline as Jemperli, received expedited approval from the FDA in April 2021 [139]. This rapid approval was only given for the treatment of dMMR endometrial malignancies; hence, it was authorized with companion diagnostic equipment called the VENTANA MMR RxDx Panel, which is used to select patients who are suitable candidates for treatment [139].

### 5.1. History and Timeline

Phase I/II and phase III clinical studies for the PD-1 inhibitor dostarlimab were underway in 2020 [140,141,142]. The manufacturer Tesaro disclosed promising early findings from the phase I/II GARNET study in 2020 [140,143]. Dostarlimab was identified as having significant potential to address a particular population of people with persistent or advanced endometrial cancer in 2020 by the GARNET study [140]. Dostarlimab was authorized in April 2021 to diagnose recurrent or advanced endometrial cancer in patients who had previously received platinum-containing chemotherapy regimens and had mismatch repair-deficient (dMMR) mutations, which are genetic defects that impair DNA repair [144]. The FDA gave dostarlimab-gxly (Jemperli, GSK) accelerated authorization in April 2021 [145]. In the multicohort, multicenter, open-label GARNET trial (NCT02715284), which comprised patients with advanced solid tumors, efficiency was assessed based on the cohort (A1) [146,147].

**Figure 3:**
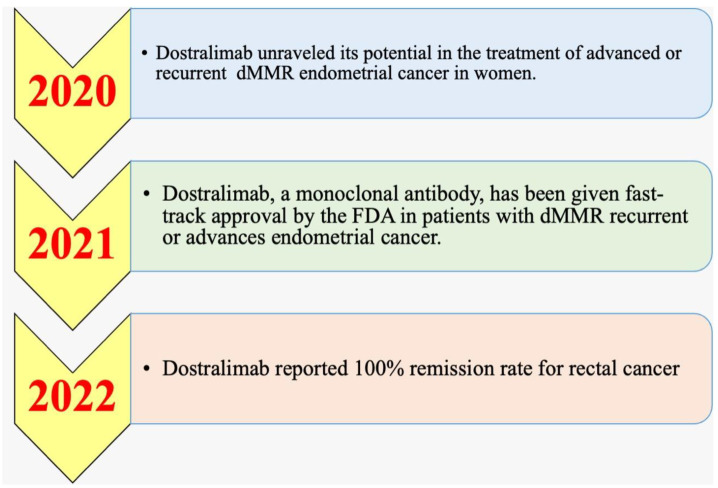
Timeline for the medicine dostarlimab, from when it first showed potential in treating certain women with EC until it was reported to have a complete remission rate for rectal cancer.

One of the latest clinical experiments on dostarlimab for rectal cancer provided a significant breakthrough in cancer therapeutics. As a “first in history” result, patients who received the experimental therapy had their cancer go away completely. There was a total of 18 participants in the study. Memorial Sloan Kettering Cancer Center in New York City treated all of them for rectal cancer. Rectal cancer was found to be locally progressed in all of the individuals. As a result, the tumors had spread only to the lymph nodes and not further into the body. Six months of treatment with dostarlimab were administered to the patients. It was carried out every three weeks for the allotted time. Cancer was screened at the end of the study and was found to be absent by physical examination, endoscopy, MRI scans, and PET scans. Dostarlimab helps the immune system recognize and eliminate cancer cells by “unmasking” them. Before participating in the clinical research, the patients received chemotherapy, radiotherapy, and invasive surgery to treat their cancer. After the experiment, they could avoid the agonizing chemotherapy and radiation treatments they had been subjected to. As of 25 months after the completion of the experiment, there were no severe post-treatment problems or symptoms of cancer recurrence. GlaxoSmithKline, a pharmaceutical corporation, funded the research. The clinical study on rectal cancer initiated in 2017 by Dr. Luis A. Diaz Jr. of the Memorial Sloan Kettering Cancer Center was the impetus for more research on the disease. Dostralimab was seen as potentially useful in the treatment of advanced or recurrent dMMR endometrial cancer in the year 2020 (Figure 3). On 17 August 2021, the FDA approved the use of the monoclonal antibody dostarlimab in patients with dMMR recurrent or advanced endometrial cancer. In 2022, announced a 100% diminution rate was announced for rectal cancer and the treatment of human cancers was revolutionized [148].

### 5.2. Pharmacodynamics and Pharmacokinetics

Dostarlimab coupled to human and cynomolgus monkey PD-1 with great affinity as per surface plasmon resonance and flow cytometry by using overexpressing recombinant PD-1 cell lines. Additionally, it is bound to the natural protein on peripheral blood mononuclear cells. The antibody was able to prevent the interaction between the receptor and PD-L1 as well as PD-L2 [149]. In a human CD4+ mixed lymphocyte-response test, dostarlimab was employed as a potent functional antagonist, which led to increased production of IL-2. In this particular test, the addition of anti-LAG3 or anti-TIM3 antibodies augmented dostarlimab’s activity level. Dostarlimab alone did not significantly stimulate the release of cytokines when it was incubated with human peripheral blood mononuclear cells [150,151]. In the fight against cancer, the immunotherapy known as dostarlimab acts as a catalyst for the endogenous anti-tumor immune response of the body. Depending on the cycle, it can be administered through intravenous infusion once every three to six weeks for 30 min [149].

Dostarlimab and other drugs that interfere with the PD-1/PD-L1 pathway disable a potent inhibitory immune-system response. Consequently, these drugs can potentially cause immunological-mediated adverse events, some of which can be life-threatening or even deadly. These responses can happen in any organ system and can happen at any time after commencing therapy. It is crucial to keep an eye on patients on dostarlimab treatment for signs of a more profound immune-mediated response. They should be examined and treated as soon as possible if an immune-mediated reaction is thought to be occurring [152].

The pharmacokinetics (PK) profile of dostarlimab enables an augmentation in the dosing interval from three to six weeks. Both in vitro and in vivo research conditions were used to study the pharmacodynamic action of dostarlimab. Every three weeks for the first cycle, 500 mg of dostarlimab was delivered intravenously, and mean Cmax and AUC0-tau values were 171 mcg/mL and 35,730 mcg h/mL, respectively. The mean AUC0-tau and Cmax were 95,820 mcg.h/mL and 309 mcg/mL, respectively, when 1000 mg was given every six weeks. Dostarlimab’s typical volume of distribution is 5.3 L at a steady state. Dostarlimab is thought to be broken down into smaller peptides and amino acids via catabolic pathways, although its metabolism has not yet been fully described [153]. Dostarlimab has an average terminal elimination half-life of 25.4 days and an average clearance of 0.007 L/h. There are no records of dostarlimab overdoses. Overdosage symptoms may involve considerable immune-mediated reactions and are anticipated to be congruent with dostarlimab’s adverse-impact profile [154,155]. Long half-life, little extravascular diffusion, and no influence of hepatic- or renal-function deterioration on PK are the PK features of other approved PD-1 inhibitors [41]. Time-varying medication clearance is another characteristic of the anti-PD-1 family. Nevertheless, certain variations in the PK characteristics of anti-PD-1 medications may be due to target-mediated drug disposition. PD1 inhibitor cemiplimab has a set dose schedule, whereas PD1 inhibitors nivolumab and pembrolizumab have static and body-weight dosage regimens based on their approved applications. The PK and exposure-response (ER) investigations of dostarlimab in patients with recurrent/advanced solid tumors were discussed after using data from the GARNET study [41,155]. Because dostarlimab is a monoclonal antibody, it does not serve as a substrate for cytochrome P450 or drug transporters, and it is not predicted to be a cytokine modulator. Because of these factors, there are no known instances of it engaging in interactions with other medications [156].

### 5.3. Action Mechanism

**Figure 4:**
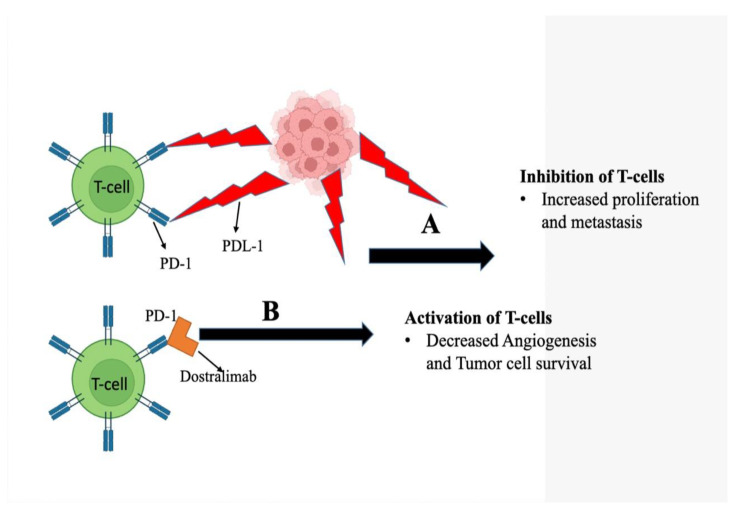
Diagram depicting the action mechanism of dostarlimab against cancer cells. The dostarlimab blocks the interaction of T cells over-expressing PD-1 protein with the PD-L1 present in cancer cells. (**A**) Cancer cells overexpress PD-L1 on their surface that interact with PD-1 of T cells. This disables the immune system from recognizing and targeting cancer cells. (**B**) Dostarlimab interacts with the PD-1 receptor on T cells, preventing their interaction with PD-L1 on cancer cells. This restores the T cells’ cytotoxic function, allowing them to detect and kill cancer cells.

A total of 13–30% of recurrent endometrial malignancies have been linked to microsatellite instability (MSI) or a mismatch repair failure (dMMR) [135,138]. The alterations in dMMR endometrial malignancies are predominantly somatic (90%). In contrast, germline mutations are involved in just 5–10% of cases [139]. Cancers that have mutations that result in dMMR can upregulate the expression of PD-1 receptor and ligands PD-L1 and PD-L2. PD-1 is present on T-cells and, when stimulated, limits their proliferation as well as the generation of cytokines. The immunological checkpoint is created as a result of the binding of these ligands to PD-1 and its subsequent downregulation of the immune response to tumors (Figure 4) [140,157]. Dostarlimab, a medication, enters the scene at this point as an option. It does this by inhibiting the receptor PD-1 and blocking the binding of receptors with PD-L1 and PD-L2. This, in turn, stimulates T cells and boosts immunity as a whole [143].

### 5.4. Immunogenicity

Compared to the incidence rates of other anti-PD-(L) 1 medications, the rate of ADA associated with dostarlimab is 2.5%. However, therapy with dostarlimab only induces a feeble immune response in a minority of cancer patients after completing one or more treatment cycles. The data of validated tests for ongoing clinical studies of dostarlimab were used to come to these conclusions on the serum ADA levels [132]. Because of the high purity of dostarlimab and the way it is delivered, there is a decreased likelihood that it may provoke an immune response. At this point, it is unknown whether preexisting ADAs or the formation of new ADAs has any bearing on the metrics of protection or effectiveness. Dostarlimab, a new anti-PD-1 antibody that is both efficacious and efficient, was found to have a negligible risk of triggering immunogenic responses in clinical trials [158].

## 6. GARNET Trial Overview

Dostarlimab (also known as TSR-042), an anti-PD-1 antibody, was evaluated in this open-label, multicenter, phase I research in patients with advanced solid tumors who have few other therapeutic options. The objective of the study was to determine whether this antibody effectively treats the ailment of participants [156]. A 6-week anti-PD-1 dosage regimen was tested in patients for the first time in the GARNET study. Based on results from the GARNET study, dostarlimab was licensed for use in adults with recurrent or advanced mismatch repair-deficient endometrial cancer that had progressed during or after treatment with a platinum-containing regimen. The research was carried out in two parts, with the first one focusing on assessing the security, pharmacokinetics, and pharmacodynamics of increasing dosages of dostarlimab. Part 1 of the study was completed first [159]. Dose escalation for dostarlimab is based on increasing weight-based dose levels and is continued until the maximum tolerated dose is reached. However, it may be discontinued at any dose up to the maximum dose of 20 milligrams per kilogram (mg/kg) in accordance with new safety and PK/PD data. Part 2 was broken down into two subparts, Part 2A (cohorts for the evaluation of fixed doses) and Part 2B (cohorts for the evaluation of variable doses) (expansion cohorts). Dostarlimab’s safety and tolerability were investigated in the trial’s Part 2A at fixed dosages of either 500 mg given every three weeks (Q3W) or 1000 mg given every six weeks. Both of these dosing schedules were followed for the duration of the research (Q6W). Dostarlimab’s efficacy and safety in the clinic were investigated in the study’s Part 2B, which focused on cohorts of patients with advanced solid tumors [143,152,160].

TSR-042 has demonstrated having potential as an anti-PD-1 treatment in ongoing clinical studies, such as FIRST (NCT03602859), RUBY (NCT03981796), and many others, where it is being examined both as a monotherapy and in combination with other anti-PD-1 therapies (Table 2).

## 7. Conclusions

In the clinical advancement of anticancer medications, immunotherapy has emerged as the next area of competition as an adjuvant treatment. As both adjuvant and neoadjuvant therapy, immunotherapy has demonstrated efficacy in the clinical diagnosis of cancer. Even though significant progress has been achieved in cancer immunotherapy, the poor response rate is difficult for cancer treatment. Over the past few years, cancer immune-checkpoint inhibitors, including PD-1 and PDL1, have checkpoint systems like the PD-1/PD-L1 pathway that have led to a clinically substantial antitumor response. Quick progress is being established in treating multiple tumor types, leading to the first FDA clearance for this newfound drug in patients with advanced melanoma based on stringent safety and efficiency requirements. PD-1 pathway inhibitors are now being studied in many cancer types and combinations with other FDA-approved therapies. Biomarker development should be accelerated because only a small number of patients will be able to benefit from this therapy and due to the likely high cost of such treatments, so it makes sense to prioritize patients who are most likely to benefit and enrich clinical trials during the FDA approval process of these novel agents.

Long-term PD-1/PD-L1 inhibition and its combination therapy may be able to manage or even cure malignant illnesses, providing new information for the treatment of cancer. Agents or treatments can modify the levels of PD-1 and PD-L1 to have an impact comparable to that of ICIs. Due to the immune system’s innate specificity, flexibility, and memory, scientists can continually and accurately eliminate cancer cells. The subsequent objective of preclinical and clinical research is to identify good combinations of PD-1/PD-L1 blockade and other possible treatments to mitigate toxic side effects, elicit powerful antitumor immune responses, and specifically eliminate cancer cells, transforming cancer into a curable and chronic disease. Therefore, in addition to initial-phase clinical testing of potential immune-modulatory medicines and innovative combinations of immune modulators and ICI with the other cancer treatments, more research is being done to uncover biomarkers linked to ICI resistance and response.

## Figures and Tables

**Table 1:** FDA-approved PD-1/PD-L1 immune inhibitors.

S. No.	Drug Name	Trade Name	Year	Target	Developer			Refrences			
1	Nivolumab	Opdivo	Melanoma (2014), NSCLC (2015), Hodgkin’s lymphoma (2016), head and neck squamous-cell cancer (2016), Hepatocellular carcer (2017), urothelial cancer (2017), CRC (2017), renal-cell cancer (2021), adenocarcinoma, esophageal cancer (2021)	PD-1	Dako Bristol–Meyers Squibb			[42,43,44]			
2	Pembrolizumab	Keytruda	Melanoma (2014), NSCLC (2015), head and neck squamous-cell cancer (2016), bladder cancer (2017), gastroesophageal cancer (2017), Hodgkin’s lymphoma (2017),	PD-1	Dako Merck					[45,46]	
3	Cemiplimab	Libtayo	Cutaneous squamous-cell cancer (2018), NSCLC (2021), basal-cell cancer (2021)	PD-1	Regeneron					[46]	
4	Dostarlimab	Jemperli	Endometrial cancer and recurrent or advanced solid tumors (2021)	PD-1	GlaxoSmithKline LLC					[41]	
5	Atezolizumab	Tecentriq	Urothelial cancer (2016), NSCLC (2016), hepatocellular melanoma (2020), carcinoma (2020), small-cell lung cancer (2021)	PD-LI	Ventana Genentech/Roche					[47]	
6	Durvalumab (MED14736)	Imfinzi	Urothelial carcinoma (2017), NSCLC (2018)	PD-LI	Ventana Medimmune/AstraZeneca				[48]		
7	Avelumab	Bavencio	Urothelial carcinoma (2017), Merkel-cell carcinoma (2017), renal-cell cancer (2019)		Merck KGaA, Darmstadt, Germany, and Pfizer				[49]		

NSCLC = Non-small-cell lung cancer.

**Table 2:** Dostarlimab clinical trials alone and with other combinations until now.

Condition or Disease	Intervention/Treatment Drug	Allocation	Phase	Estimated Enrollment	ClinicalTrials.gov Identifier:	Duration
Breast cancer	Dostarlimab, dostarlimab + combinations	N/A	Phase II	4000 participants	NCT01042379 Recruiting	1 March 2010 December 2031
Neoplasms	Dostarlimab	Non-Randomized	Phase I	740 participants	NCT02715284 Recruiting	7 March 2016 30 July 2024
Neoplasms	Dostarlimab, dostarlimab + combinations	Non-Randomized	Phase I	369 participants	NCT02817633 Recruiting	8 July 2016 3 October 2024
Advanced solid tumors	Dostarlimab	Non-Randomized	Phase I	111 participants	NCT03250832 Active, not recruiting	8 August 2017 14 November 2022
Neoplasms	Niraparib, pembrolizumab, TSR-042 (dostarlimab)	Non-Randomized	Phase II	53 participants	NCT03308942 Completed	29 September 2017 31 August 2021
Advanced cancer, neoplasms, metastatic cancer, solid tumor, lung carcinoma	Niraparib, TSR-042, carboplatin–paclitaxil, bevacizumab, TSR-022, carboplatin–pemetrexed, carboplatin–nab-paclitaxe	Non-Randomized	Phase I	58 participants	Active, not recruiting NCT03307785	12 October 2017 29 April 2022
Endometrial cancer	Niraparib, dostarlimab	N/A	Phase II	51 participants	NCT03016338 Active, not recruiting	6 November 2017 December 2023
Ovarian neoplasms, ovarian cancer	Niraparib, dostarlimab (TSR-042).	Randomized	Phase III	1405 participants	NCT03602859 (First) Active, not recruiting	11 October 2018 22 June 2026
Ovarian neoplasms	Niraparib, TSR-042, bevacizumab, carboplatin, paclitaxel	Randomized	Phase I Phase II	125 participants	NCT03574779 Recruiting	15 November 2018 31 March 2026
Neoplasms	Dostarlimab, carboplatin, paclitaxel, niraparib	N/A	Phase III	740 participants	NCT03981796 (Ruby) Recruiting	11 June 2019 29 December 2026
Cervical cancer, advanced cancer	Dostarlimab	Randomized	Phase II	132 participants	NCT03833479 Recruiting	28 June 2019 December 2024
Ovarian neoplasms	Dostarlimab, niraparib	N/A	Phase II	41 participants	NCT03955471 (Moonstone) Terminated	3 October 2019 12 January 2022
Endometrial cancer	Dostarlimab Radiation: brachytherapy Procedure: endometrial, blood draw for immune-response biopsy	N/A	Phase I	12 participants	NCT03955978 Recruiting	15 October2019 31 October 2024
Advanced mismatch repair-deficient solid tumors	Dostarlimab, capecitabine or 5-FU Radiation: intensity modulated radiation therapy	Non-randomized	Phase II	30 participants	NCT04165772 Recruiting	11 December 2019 30 November 2025
Localized unresectable, adult primary liver cancer, adult primary liver cancer, advanced adult primary liver cancer	TSR-022 and dostarlimab	N/A	Phase II	42 participants	NCT03680508 Recruiting	19 December 2019 October 2023
Melanoma stage III Melanoma stage IV	Dostarlimab (single), dostarlimab and TSR-022 (combination)	Randomized	Phase II	56 participants	NCT04139902 Recruiting	30 April 2020 October 2027
Endometrial carcinoma, ovarian carcinoma	Niraparib (single), niraparib + ostarlimab drugs	Randomized	Phase II	Phase 3 196 participants	NCT03651206 Recruiting	15 July 2020 June 2025
Head and neck cancer	Dostarlimab, niraparib	N/A	Phase II	23 participants	NCT04313504 Recruiting	4 November 2020 1 June 2027
Ovarian cancer	Dostarlimab, niraparib, pegylated liposomal doxorubicin, paclitaxel, bevacizumab, gemcitabine, topotecan	Randomized	Phase III	427 participants	NCT04679064 Recruiting	1 December 2020 1 January 2025
Breast cancer	Dostarlimab, niraparib	Randomized	Phase II	62 participants	NCT04584255 Recruiting	18 December 2020 17 July 2029
Neuroendocrine carcinomas	Dostarlimab, niraparib	N/A	Phase II	48 participants	NCT04701307 Recruiting	1 February 2021 30 May 2025
Sarcoma, clear cell	Dostarlimab	N/A	Phase II	16 participants	NCT04274023 Recruiting	19 February 2021 1 May 2024
Endometrial cancer	Dostarlimab, intensity-modulated radiation therapy	N/A	Phase II	31 participants	NCT04774419 Recruiting	2 April 2021 February 2023
Breast cancer	Niraparib, dostarlimab Radiation: radiation therapy	N/A	Phase II	32 participants	NCT04837209 Recruiting	21 July 2021 1 December 2029
HRD cholangiocarcinoma metastatic cancer	Niraparib, dostarlimab	N/A	Phase II	47 participants	NCT04895046 Recruiting	11 October 2021 September 2023
RCA-mutated pancreas, breast, ovarian cancer	Dostarlimab, niraparib	N/A	Phase I	18 participants	NCT04673448 Recruiting	18 October 2021 10 March 2026
Ovarian cancer	Niraparib, dostarlimab	Non-randomized	Phase II	100 participants	NCT05126342 Not yet recruiting	1 May 2022 1 November 2026
Colon cancer, DMMR colorectal cancer	Dostarlimab	N/A	Phase II	29 participants	NCT05239546 Not yet recruiting	June 2023 June 2029

N/A: not available. Source: https://clinicaltrials.gov (accessed on 14 September 2022).

## Data Availability

Not applicable.

## Abbreviations

ADA	Anti-drug antibodies
ALK	Anaplastic lymphoma kinase
APC	Antigen-presenting cell
ATG	Antithymocyte globulin
BCG	Bacille Calmette–Guerin
CD	Cluster of differentiation
CRC	Colorectal cancer
DCs	Dendritic cells
DLBCL	Diffuse large B-cell lymphoma
dMMR	Deficient mismatch repair
DLs	Dose levels
EGFR	Epidermal growth-factor receptor
EMT	Epithelial-to-mesenchymal transition
ERK	Extracellular signal-regulated kinase
ER	Estrogen receptor
FDA	Food and Drug Administration
FGFR	Fibroblast growth-factor receptor
FOX	Forkhead box protein
GIST	Gastrointestinal stromal tumors
GCB	Germinal center B cell
GSK	GlaxoSmithKline
GLI	Glioma-associated oncogene homolog
Hh	Hedgehog
HNSCC	Head and neck squamous-cell carcinoma
HL	Hodgkin’s lymphoma
ICIs	Immune-checkpoint inhibitors
IFN	Interferon gamma
IL	Interleukin
IRF	Interferon regulatory factor
JAK	Janus kinase
Kras	Kirsten rat sarcoma viral oncogene
LAG	Lymphocyte-activation gene
MRI	Magnetic resonance imaging
MTD	Maximum tolerated dose
MCF	Michigan Cancer Foundation-7
MHC	Major histocompatibility complex
MET	Microenvironment
MSI-H	Microsatellite instability—high
MAPK	Mitogen-activated protein kinase
NSCLC	Non-small-cell lung cancer
NOTCH	Neurogenic locus notch homolog protein 1
TNF	Tumour necrosis factor
NFAT	Nuclear factor of activated T cells
OS	Overall survival
PDAC	Pancreatic ductal adenocarcinoma
PBMC	Peripheral blood mononuclear cells
PTEN	Phosphatase and tensin homolog
PKC	Protein kinase C
PET	Positron emission tomography
PD	Programmed cell death
PD-L	Programmed cell-death ligand
RAS	Rat sarcoma
RCC	Renal-cell carcinoma
STAT	Signal transducer and activator of transcription
SMO	Smoothened
TCR	T-cell receptor
TIM-3	T-cell immunoglobulin and mucin-domain-containing-3
TLR	Toll-like receptor
TNBC	Triple-negative breast cancer
TILs	Tumour-infiltrating lymphocytes
WNT	Wingless-related integration site
